# Physical activity counselling in women with HIV/AIDS and suicidal ideation: a secondary analysis of a real-world intervention in Ugandan HIV counselling centres

**DOI:** 10.11604/pamj.2023.45.70.40093

**Published:** 2023-05-31

**Authors:** Davy Vancampfort, James Mugisha, Simon Rosenbaum, Tine Van Damme

**Affiliations:** 1 KU Leuven Department of Rehabilitation Sciences, Leuven, Belgium,; 2 University Psychiatric Centre KU Leuven, Leuven-Kortenberg, Belgium,; 3 Department of Sociology and Social Administration, Kyambogo University, Kampala, Uganda,; 4 School of Psychiatry, University of New South Wales, Sydney, New South Wales, Australia

**Keywords:** Alcohol, depression, physical activity, suicide

## Abstract

**Introduction:**

people with HIV/AIDS have higher rates of suicidal ideation than the general population. Consequently, HIV counselling settings should implement suicide risk reduction initiatives. Physical activity (PA) counselling could be a relevant add-on intervention. The aim of this secondary analysis from a single-arm pre- and post-study exploring the efficacy of PA counselling for HIV/AIDS patients with mental health problems was to investigate the efficacy of PA counselling on reducing suicidal ideation.

**Methods:**

out of 41 participants in an 8-week PA counselling intervention, 15 participants reported suicidal ideation. These 15 (15♀, median age=42 years, interquartile range=24 years) participants completed the Patient Health Questionnaire-9 (PHQ-9), Alcohol Use Disorder Identification Test -10 (AUDIT-10), and the Simple Physical Activity Questionnaire (SIMPAQ) pre- and immediately post-intervention.

**Results:**

the prevalence of suicidal ideation (PHQ-9 item 9≥1) dropped to 20% post-intervention, i.e. only three patients with HIV still reported suicidal ideation. Also, following the intervention significant (P<0.05) increases in walking and incidental PA (SIMPAQ) levels, and reductions in depressive and alcohol abuse symptoms were observed.

**Conclusion:**

our data demonstrate that PA counselling might be promising in reducing suicidal ideation in most HIV patients in low-resourced settings. Randomized controlled trials are warranted to confirm these beneficial findings.

## Introduction

Mental health and HIV/AIDS are strongly interlinked. One is affecting [1] and predisposing to [2,3] the other. For example, mental health issues can arise as a consequence of an HIV infection, the experienced burden, associated stigma, side effects of antiretroviral therapy and presence of associated co-morbidities such as opportunistic infections [4,5]. Mental illness is also a risk factor for contracting HIV/AIDS while impeding management due to poor insight and judgment [6]. Compared to the general population, people living with HIV/AIDS have a 100-fold higher risk of completed suicide [7]. This supports the strong need for HIV/AIDS counselling services to integrate mental health support, including suicidal risk prevention. In particular, this is essential in sub-Saharan African countries where the highest burden of HIV/AIDS is situated and mental health services are over-demanded or even entirely lacking [8]. While in countries, such as Uganda, most care providers in health centers are knowledgeable about suicide and associated risk factors, they report challenges in assessing and managing individuals with suicide risk [9,10]. For example, a recent study in 7,958 health facilities across seven low-income countries demonstrated that amitriptyline, an antidepressant classified as essential by the World Health Organization (WHO) [11], was only available in only 8% of health facilities on the day of assessment [12]. Therefore, feasible and scalable non-pharmacological interventions addressing suicidal ideation are urgently required. Although cognitive behavioral therapy is effective in reducing the mental health burden in people with HIV/AIDS [13], a key gap is the lack of integration of psychotherapy interventions as a part of accessible evidence-based care [14]. One of the reasons is that most local health centers in low-income countries have very few trained mental health specialists and burdened staff [15].

Recently, the role of lifestyle psychiatry has been acknowledged as a promising non-pharmacological intervention for reducing the burden of poor mental health [16,17]. Physical activity is, for example, a low-cost lifestyle intervention which demonstrated to be promising in reducing suicidal risk [18]. The few existing trials were however executed in high-income countries. Real-world interventions in low-income countries are lacking. Exploring the effectiveness and efficacy in real-world interventions delivered by non-experts is important, particularly because drop-out rates from physical activity interventions are as high as 20 to 30% in people experiencing mental health problems [19-22]. Therefore, in recent years, several calls were made to explore novel approaches to reduce the mental health burden via lifestyle interventions [17,23]. There is strong evidence that autonomy-supportive environments increase adherence towards an active lifestyle and reduce drop-out rates from physical activity programs in vulnerable populations [24-26]. Within autonomy-supportive environments, autonomous (e.g. enjoyment and/or personal value) instead of controlled reasons (e.g. guilt and/or extrinsic reinforcements) for behavior change are facilitated [27,28].

In a pilot study [29], we demonstrated that autonomy-supportive physical activity counselling in inactive people with HIV/AIDS and a co-morbid mental disorder is efficacious in increasing health-related quality of life and physical activity levels. The aim of this secondary analysis of our pilot study was to explore the efficacy of physical activity counselling in reducing levels of suicidal ideation in patients with HIV/AIDS and a co-morbid mental disorder living in a remote Ugandan fishing community. A secondary aim of the current study was to explore whether any changes in suicidal ideation were accompanied by changes in depression, alcohol abuse and physical activity levels. We hypothesize that physical activity counselling will reduce suicidal ideation and is accompanied by reductions in depression and alcohol abuse and increases in physical activity levels.

## Methods

**Study design:** this is a secondary analysis of a pre-test/post-test study without a control group [29].

**Study setting:** this study took place in May-June 2018 in two HIV counselling centers in fishing communities in Buikwe District in Central-Uganda.

**Study population:** in this pilot study, all people with a diagnosis of HIV/AIDS: (a) who received antiretroviral therapy in one of two selected health centers in fishing communities in Buikwe District in Central-Uganda, (b) who were not complying with the physical activity recommendation of 150 min of moderate to vigorous physical activity per week as assessed with the Physical Activity Vital Sign [30], and (c) who had either major depressive disorder as assessed with the Patient Health Questionnaire -9 [31] or alcohol use disorder as assessed with the Alcohol Use Disorder Test - 10 [32] were invited to participate in an 8-week (once per week) physical activity counselling intervention which was based on the principles of the self-determination theory (SDT) and motivational interviewing framework. In total 81 of 256 screened participants did not comply with the physical activity recommendations (31.6%). The prevalence of major depressive disorder was 11.7% (30/256), while the prevalence of alcohol use disorder was 10.5% (27/256). Of these 256, 45 fulfilled the criteria and were included in an 8-week physical activity counselling intervention. Four participants dropped out [29]. Of the 41 participants who completed the intervention, 15 reported suicidal ideation at baseline. In agreement with recent Ugandan studies [33,34], suicidal ideation was in this secondary analysis considered present when participants scored 1 or higher on item 9 of the Patient Health Questionnaire-9 (PHQ-9) [35]. The 15 participants with suicidal ideation who completed the study were included in the current secondary analysis.

**Study sampling:** the two HIV counselling centers were via the Health District Office randomly selected using a sealed envelope procedure.

**Study intervention:** the counselling could consist of either 15 to 20 min individual counselling for participants living in remote areas or either one hour to one hour and 30 min group counselling for those living near the health centers. To create an autonomy-supportive environment, the clinical officer providing the counselling promoted in each participant a sense of ownership over their daily life activities that lead to internal perceived locus of control. This involved (a) building sustainable knowledge that supported informed choices, by using neutral language during interpersonal communication (e.g., “may” and “could”, and not “should” or “must”); (b) encouraging choice and self-initiation while the use of pressure, demands, prescriptions, and extrinsic rewards were avoided; (c) providing patients with a menu of options and a variety of avenues for implementing physical activity in their daily life; (d) supporting a chosen activity by presenting clear contingencies between the activity and the desired mental, physical or social health outcome; (e) encouraging patients to build and explore congruence between their values and goals in life, and their lifestyle (e.g., becoming an active contributor to the family or society again) and (f) giving informational positive feedback, acknowledging that the feeling of competence grows from feedback inherent to the activity.

**Study variables:** patients were asked whether they had a paid job (yes versus no). Age, gender, and the presence of chronic somatic comorbidities (yes versus no and which condition) were self-reported and, when possible, confirmed via the medical files. Multimorbidity was considered being present if two or more chronic somatic comorbidities were reported. Use of psychopharmacotherapy was self-reported and, when possible, confirmed via the medical files. For calculating the body mass index (BMI), weight was measured in light clothing to the nearest 0.1kg using a SECA beam balance scale, and height to the nearest 0.1cm using a wall-mounted stadiometer. Participants were assessed pre- and post-intervention using the Simple Physical Activity Questionnaire (SIMPAQ) [36], the Patient Health Questionnaire-9 (PHQ-9) [31], and the Alcohol Use Disorders Identification Test (AUDIT) [32].

**Patient Health Questionnaire-9 (PHQ-9)** [35]**:** patients completed the Luganda version of the PHQ-9 [35] pre- and post-intervention. The nine items are based directly on the nine diagnostic criteria for major depressive disorder in the DSM-IV (American Psychiatric Association, 1994). For each item, the individual is asked to rate the severity of his or her symptoms over the past two weeks, by providing a score on a Likert scale with symptoms rated as 0 (not at all), 1 (several days), 2 (more than half the days) and 3 (nearly every day). Higher scores indicate more severe symptoms of depression. Suicidal ideation was considered present when the participants scored 1 or higher on item 9 evaluating suicidal ideation in the past two weeks. The PHQ-9 has a good internal consistency reliability, test-retest reliability, and construct validity and has been used previously in people with mental health problems in Uganda [37,38]. The Luganda version of the questionnaire was interviewer-administered.

**Alcohol Use Disorders Identification Test (AUDIT):** to assess the presence of alcohol use disorders in this study, we used the AUDIT [32], which was developed by the World Health Organization (WHO) as a simple method of screening for alcohol use disorders. The AUDIT comprises three domains: hazardous alcohol use (frequency of drinking, typical quantity, and frequency of heavy drinking), dependence symptoms (impaired control over drinking, increased salience of drinking, and morning drinking), and harmful alcohol use (guilt after drinking, blackouts, alcohol-related injuries, and other concerns about drinking). The AUDIT was adapted for local use by using of pictures and local terms for standard alcohol units. In accordance with previous research in Uganda, a score of 8 or more was considered to be a positive screening result [39]. The Luganda version of the questionnaire was interviewer-administered.

**Simple Physical Activity Questionnaire (SIMPAQ)** [36]**:** physical activity was assessed pre- and post-intervention with the Luganda version of the SIMPAQ [36]. The SIMPAQ [36] is a 5-item clinical tool to assess physical activity among populations at high risk for sedentary behavior. It uses an interview format to estimate time spent in bed (min/day), time spent sedentary during waking hours (min/day), time spent napping (min/day), time spent walking (min/day), time spent in structured exercise (min/day), and time spent in incidental or non-structured physical activity (min/day) during the past week. The sum of the hours recorded in the SIMPAQ items should add to approximately 24-hours, providing interviewers with an opportunity to clarify if significant under or over-reporting has occurred by the participants (e.g. total of <18 hours or >30 hours accounted for). Previous research in Uganda demonstrated the questionnaire is reliable [40], while validity has been demonstrated in a 23-country validation study [41]. In this study, we used time spent walking (min/day), time spent in structured exercise (min/day), and time spent in incidental or non-structured physical activity (min/day) during the past week as a measure of proxy for physical activity. Digging was considered a structured activity of moderate to vigorous intensity and therefore presented as an example under structured exercise in this fishing community. The Luganda version of the questionnaire was interviewer-administered.

**Study procedure:** in the period May-June 2018 all patients with HIV attending one of two randomly selected HIV counselling centers in fishing communities in Buikwe District in Central-Uganda were invited to participate in the baseline screening. Forty-five of 256 screened patients were physically inactive and self-reported either major depressive disorder and/or alcohol use disorder and were invited to participate in the intervention. Four dropped out. Of the 41 remaining participants who completed the original study, 15 reported suicidal ideation and were included in this secondary analysis. All 15 were women and therefore no sex-related analyses were performed.

**Statistical analyses:** data were tested for normality using the Shapiro-Wilks test and found not to be normally distributed. Changes in outcome measures were evaluated using Wilcoxon signed-rank tests (P< 0.05). SPSS version 28.0 (SPSS Inc., Chicago) was used for all data analyses.

**Ethical considerations:** written informed consent was obtained from all the participants, with illiterate participants providing consent via a fingerprint. The pilot study was approved by the ethical committee of Mengo Hospital.

**Funding:** this research was funded by KU Leuven Global Minds.

**Data availability:** the data can be obtained from the corresponding author upon reasonable request.

## Results

**Participants:** of 41 (33 women) patients with HIV/AIDS completing the pilot study, fifteen women reported suicidal ideation at baseline. These 15 [median (interquartile range, IQR) age=42.0 (24.0)] were included in this secondary analysis study. Four patients had a paid job, no single patient was taking psychotropic medication. The median body mass index was 23.3 (IQR=4.0). One participant smoked and reported to smoke on average one cigarette per day. Two patients complained of leg pain, three patients reported chronic asthma, three chronic cardiovascular symptoms, four chronic low back pain and four chronic joint pain. Five patients reported multi-morbidity, i.e. two or more chronic conditions.

**Changes in clinical variables following the 8-week physical activity counselling:** between baseline and 8 weeks of counselling, the prevalence of suicidal ideation reduced from 100 to 20.0% (P=0.001). Only three participants reported suicidal ideation post-intervention. Other changes in time of the clinical characteristics are presented in Table 1. Significant reductions in PHQ-9 (P<0.001) and AUDIT-10 (P=0.036) scores were observed, and increases in the time spent walking (P=0.002), and in incidental physical activity (P=0.006). No changes in the structured exercise levels were found (P=0.89) (Table 1).

**Table 1:** changes in clinical variables in HIV/AIDS patients with suicidal ideation (n=15) following 8 weeks of physical activity counselling

Variables	Pre-test Median (interquartile range) or %	Post-test Median (interquartile range) or %	P
Suicidal ideation (n, prevalence, %)	15/15 (100%)	3/15 (20%)	0.001*
PHQ-9 total score	7.0 (4.0)	3.0 (4.0)	<0.001*
AUDIT-10 total score	1.0 (4.0)	0.0 (0.0)	0.036*
SIMPAQ walking (min/day)	2.0 (1.0)	60.0 (146.0)	0.002*
SIMPAQ exercise (min/day)	0.0 (10.0)	0.0 (3.0)	0.89
SIMPAQ incidental (min/day)	27.0 (20.0)	160.0 (180.0)	0.006*

* Significant when P<0.05. AUDIT-10 = Alcohol use Disorder Test - 10, PHQ-9= Patient Health Questionnaire, SIMPAQ=Simple Physical Activity Questionnaire.

## Discussion

To the best of our knowledge, the current study is the first to indicate that in a remote fishing community an 8-week physical activity counselling program using a SDT and motivational interviewing framework reduces levels of suicidal ideation. An interesting observation is that only women with HIV/AIDS reported suicidal ideation in the current study. Recently, a gender-based meta-analysis found that the prevalence of suicide/suicidal ideation is higher among women compared to men [42]. Therefore, calls were made for health planners and policymakers to develop suicide-prevention strategies targeting women [42].

Our study adds to the existing literature that lifestyle interventions might be a potential strategy to reduce suicide risk in low-resourced settings, and in particular for women. A secondary finding was that in the fifteen included patients with suicidal ideation the levels of depression and alcohol abuse also reduced, and levels of physical activity increased. One of the potential underlying mechanisms for the reduced suicidal ideation therefore might be these reductions in symptomatology, which are known risk factors for suicidal risk [43-46]. There are several neurobiological and psychosocial pathways that could explain the observed reduction in suicidal ideation and depression associated with being more physically active. For example, neurobiological changes such as an increased cerebral blood flow and changes in peripheral biomarkers such as an increase in circulating neurotrophic growth factors, and anti-inflammatory markers have been reported before [47]. From a psychosocial perspective, physical activity provides people with an opportunity for social interaction (relatedness), independence (autonomy) when recovering, and mastery in the physical domain (self-efficacy and perceived competence) [48]. For the latter, being able to reduce stress levels and improve resilience through being active might be one of the mechanisms that could clarify the lower alcohol abuse levels. People with HIV/AIDS often try to cope with daily life stressors and challenges via excessive alcohol abuse, mainly in fishing communities in Uganda [49].

**Limitations and future research:** the findings of the present study should however be interpreted with caution due to some methodological limitations. First, although suicidal ideation reported via item 9 of the PHQ-9 is a robust predictor of suicide attempts and deaths regardless of age, and this for increased risk for two years [50], it should be noted that the PHQ-9 was designed to screen for depression and assess its severity, not to assess risk for suicide. Second, the one-group quasi-experimental research design employed in this study limits conclusions and generalizability. Third, physical activity was only measured with a self-report questionnaire, which is prone to both systematic and random errors [51]. Fourth, no follow-up was done after the counselling cessation.

## Conclusion

Despite the reported limitations, it can be concluded that in the majority of patients with HIV/AIDS and a co-morbid mental disorder, suicidal ideation reduced following a physical activity counselling program based on SDT principles and a motivational interviewing framework. Reductions in suicidal ideation are accompanied with reductions in depression and alcohol abuse and increases in physical activity levels. Benefits can be obtained within 8 weeks and in challenging low-resourced settings such as in the Ugandan fishing community in our study.

### What is known about this topic

- *Compared to the general population, people living with HIV/AIDS have a 100-fold higher risk of completed suicide;*
- *HIV counselling centers are knowledgeable about suicide and associated risk factors, but report challenges in assessing and managing individuals with suicide risk;*
- *Physical activity is a low-cost lifestyle intervention which could be promising in reducing suicidal risk*.

### What this study adds

- *In the majority of patients with HIV/AIDS and a co-morbid mental disorder, suicidal ideation reduces following a physical activity counselling program;*
- *The self-determination theory principles and a motivational interviewing framework can be successfully implemented in physical activity interventions in low-resourced settings;*
- *Reductions in suicidal ideation are accompanied with reductions in depression and alcohol abuse*.
